# *Taenia solium* Tapeworm Infection, Oregon, 2006–2009

**DOI:** 10.3201/eid1706.101397

**Published:** 2011-06

**Authors:** Seth O’Neal, John Noh, Patricia Wilkins, William Keene, William Lambert, James Anderson, Jenifer Compton Luman, John Townes

**Affiliations:** Author affiliations: Oregon Health & Science University, Portland, Oregon, USA (S. O’Neal, W. Lambert, J. Anderson, J. Compton Luman, J. Townes);; Centers for Disease Control and Prevention, Atlanta, Georgia, USA (J. Noh, P. Wilkins);; Oregon Department of Human Services, Portland (W. Keene)

**Keywords:** cysticercosis, neurocysticercosis, Taenia solium, epidemiology, screening, Oregon, parasites, Hispanic, research

## [MEDSCAPECME LOGO]

Medscape, LLC is pleased to provide online continuing medical education (CME) for this journal article, allowing clinicians the opportunity to earn CME credit.

This activity has been planned and implemented in accordance with the Essential Areas and policies of the Accreditation Council for Continuing Medical Education through the joint sponsorship of Medscape, LLC and Emerging Infectious Diseases. Medscape, LLC is accredited by the ACCME to provide continuing medical education for physicians.

Medscape, LLC designates this Journal-based CME activity for a maximum of 1 *AMA PRA Category 1 Credit(s)*^TM^. Physicians should claim only the credit commensurate with the extent of their participation in the activity.

All other clinicians completing this activity will be issued a certificate of participation. To participate in this journal CME activity: (1) review the learning objectives and author disclosures; (2) study the education content; (3) take the post-test and/or complete the evaluation at www.medscape.org/journal/eid; (4) view/print certificate.

**Release date: May 23, 2011; Expiration date: May 23, 2012**

## Learning Objectives

Upon completion of this activity, participants will be able to:

- Describe the epidemiology of cysticercosis in Oregon as based on a surveillance study
- Describe morbidity and mortality associated with cysticercosis as based on that surveillance study
- Describe goals of public health interventions for cysticercosis in Oregon as based on that study

## MEDSCAPE CME Editor

**Karen Foster, **Technical Writer/Editor, *Emerging Infectious Diseases. Disclosure: Karen Foster has disclosed no relevant financial relationships*.

## MEDSCAPE CME AUTHOR

**Laurie Barclay, MD, **freelance writer and reviewer, Medscape, LLC. *Disclosure: Laurie Barclay, MD, has disclosed no relevant financial relationships.*

## AUTHORS

*Disclosures:* ***Seth O’Neal, MD; John Noh; Patricia Wilkins, PhD; William Keene, PhD, MPH; William Lambert, PhD; James Anderson, MD; Jenifer Compton Luman, MD;*** *and* ***John Townes, MD,*** *have disclosed no relevant financial relationships.*
