# Supplementary material for: Taenia solium Tapeworm Infection, Oregon, 2006–2009
Source: Emerg Infect Dis. 2011 Jun;17(6):1030–6. doi: 10.3201/eid1706.101397 (PMC3320238; doi:10.3201/eid1706.101397)
Supplement: Technical Appendix — Interview tool used to gather demographic, clinical, and epidemiologic data from study participants. [file 10-1397-Techapp_2p.pdf]

Article DOI: 10.3201/eid1706.101397

Taenia solium infection, Oregon, 2006–2009

Technical Appendix

Interview tool used to gather demographic, clinical, and epidemiologic data from study participants.

Cysticercosis

COUNTY

FOR STATE USE ONLY #
☐ confirmed
☐ presumptive
___/___/___ case report
___/___/___ interstate

Use Taeniasis form for tapeworms. Date investigation initiated: ____ /____ /____

CASE IDENTIFICATION

Name ______________________ (LAST, first, initials) (a.k.a.) Phone(s) ______________ (indicate home (H); work (W); message (M))

Address ______________________ Street City County Zip

e-mail address ______________ language spoken ______________

ALTERNATE CONTACT ☐ Parent ☐ Spouse ☐ Household Member ☐ Friend ☐ ______________

Name ______________ Phone(s) ______________ (indicate home (H); work (W); message (M))

Address ______________ Street City Zip

SOURCES OF REPORT *(check all that apply)*
☐ Lab ☐ Infection Control Practitioner
☐ Physician ☐ ______________

Name ______________

Phone ______________ Date ___/___/___ *(first report)*

Primary M.D. ______________ *(if different)*

Phone ______________ OK to talk to patient? ☐

DEMOGRAPHICS

SEX
☐ female ☐ male

DATE OF BIRTH ____/____/____ (m d y)

or, if unknown, AGE ______

HISPANIC ☐ yes ☐ no ☐ unknown

RACE
☐ White ☐ American Indian
☐ Black ☐ Asian/Pacific Islander
☐ unknown ☐ refused to answer
☐ other ______________

Worksites/school/day care center/______________

Occupations/grade/______________

BASIS OF DIAGNOSIS

CLINICAL DATA

Symptoms of neurocysticercosis: ☐ yes ☐ no ☐ unk
if yes, ONSET on ____/____/____ (m d y)

Check all that apply:

seizure	☐ yes	☐ no	☐ unk
chronic/recurrent headaches	☐ yes	☐ no	☐ unk
focal weakness	☐ yes	☐ no	☐ unk
cognitive impairment	☐ yes	☐ no	☐ unk
vision changes	☐ yes	☐ no	☐ unk

☐ other ______________

LABORATORY DATA

☐ Serum EITB assay for cysts ☐ positive ☐ negative
Lab ______________ Date ___/___/___

☐ Pathologic specimen confirming *T. solium* cyst ☐ positive ☐ negative
Lab ______________ Date ___/___/___

☐ Radiographic imaging ☐ CT head ☐ MRI head ☐ Other
Result ______________

Imaging Facility ______________ Date ___/___/___

RISK FACTORS

Birthplace outside of US? ☐ yes ☐ no ☐ unk
If yes, specify country ______________

Travel or residence outside of US? ☐ yes ☐ no ☐ unk
If yes, specify countries, year and length of stay
____
____
____

Repeated exposure to human feces? ☐ yes ☐ no ☐ unk
If yes, specify
☐ daycare
☐ healthcare worker
☐ male homosexual contact
☐ other ______________

OUTCOME

Hospitalized: ☐ yes ☐ no ☐ unk
name of hospital ______________
date of admission ____/____/____ date of discharge ____/____/____
Discharged to long-term care facility: ☐ yes ☐ no ☐ unk
Outcome: ☐ survived ☐ died ☐ unk date of death ____/____/____

EPI-LINKAGE

Is the patient...
associated with a known outbreak of cysticercosis? ☐ yes ☐ no ☐ unk
a close contact of a confirmed or presumptive case of taeniasis? ☐ yes ☐ no ☐ unk
Has the above case been reported? ☐ yes ☐ not yet
Specify nature of contact: ☐ household ☐ sexual ☐ daycare ☐ other
If yes to any questions, specify relevant names, dates, places, etc.

SCREENING

Symptoms of taeniasis: ☐ yes ☐ no ☐ unk
if yes, ONSET on ____/____/____ (m d y)

Check all that apply:

abdominal pain	☐ yes	☐ no	☐ unk
abdominal distension	☐ yes	☐ no	☐ unk
diarrhea	☐ yes	☐ no	☐ unk
saw worm segments in feces	☐ yes	☐ no	☐ unk

LABORATORY DATA

☐ Stool microscopy ☐ *Taenia solium* ☐ *Taenia saginatta* ☐ *Taenia* species
Lab ______________ Date ___/___/___
☐ Initial stool coproantigen ☐ positive ☐ negative
Lab ______________ Date ___/___/___
☐ Serum EITB assay for *T. solium* tapeworm ☐ positive ☐ negative
Lab ______________ Date ___/___/___

PATIENT'S NAME

CASE-CONTACT MANAGEMENT AND FOLLOW-UP

Name ______ Age ______ Relation to Case ______ By proxy ☐ yes ☐ no Proxy name ______

NEUROCYSTICERCOSIS SCREENING
if yes to any recommend medical evaluation of neurocysticercosis.

	yes	no	unk	date
seizure	☐	☐	☐	___/___/___
cognitive impairment	☐	☐	☐	___/___/___
chronic/recurrent headaches	☐	☐	☐	___/___/___
focal weakness	☐	☐	☐	___/___/___
vision changes	☐	☐	☐	___/___/___
unexplained neuro deficit	☐	☐	☐	___/___/___

TAENISIS SCREENING
Saw worm segments in feces ☐ yes ☐ no ___/___/___
Stool microscopy ☐ *Taenia solium* ☐ *Taenia saginatta* ☐ *Taenia* species
Lab ______ Date ___/___/___
Initial stool coproantigen ☐ positive ☐ negative
Lab ______ Date ___/___/___
Serum EITB assay for *T. solium* tapeworm ☐ positive ☐ negative
Lab ______ Date ___/___/___

Comments

Stool Sample Collected ☐ yes ☐ no ☐ unk
Medical Evaluation ☐ yes ☐ no ☐ unk
Education Provided ☐ yes ☐ no ☐ unk

Name ______ Age ______ Relation to Case ______ By proxy ☐ yes ☐ no Proxy name ______

NEUROCYSTICERCOSIS SCREENING
if yes to any recommend medical evaluation of neurocysticercosis.

	yes	no	unk	date
seizure	☐	☐	☐	___/___/___
cognitive impairment	☐	☐	☐	___/___/___
chronic/recurrent headaches	☐	☐	☐	___/___/___
focal weakness	☐	☐	☐	___/___/___
vision changes	☐	☐	☐	___/___/___
unexplained neuro deficit	☐	☐	☐	___/___/___

TAENISIS SCREENING
Saw worm segments in feces ☐ yes ☐ no ___/___/___
Stool microscopy ☐ *Taenia solium* ☐ *Taenia saginatta* ☐ *Taenia* species
Lab ______ Date ___/___/___
Initial stool coproantigen ☐ positive ☐ negative
Lab ______ Date ___/___/___
Serum EITB assay for *T. solium* tapeworm ☐ positive ☐ negative
Lab ______ Date ___/___/___

Comments:

Stool Sample Collected ☐ yes ☐ no ☐ unk
Medical Evaluation ☐ yes ☐ no ☐ unk
Education Provided ☐ yes ☐ no ☐ unk

Name ______ Age ______ Relation to Case ______ By proxy ☐ yes ☐ no Proxy name ______

NEUROCYSTICERCOSIS SCREENING
if yes to any recommend medical evaluation of neurocysticercosis.

	yes	no	unk	date
seizure	☐	☐	☐	___/___/___
cognitive impairment	☐	☐	☐	___/___/___
chronic/recurrent headaches	☐	☐	☐	___/___/___
focal weakness	☐	☐	☐	___/___/___
vision changes	☐	☐	☐	___/___/___
unexplained neuro deficit	☐	☐	☐	___/___/___

TAENISIS SCREENING
Saw worm segments in feces ☐ yes ☐ no ___/___/___
Stool microscopy ☐ *Taenia solium* ☐ *Taenia saginatta* ☐ *Taenia* species
Lab ______ Date ___/___/___
Initial stool coproantigen ☐ positive ☐ negative
Lab ______ Date ___/___/___
Serum EITB assay for *T. solium* tapeworm ☐ positive ☐ negative
Lab ______ Date ___/___/___

Comments:

Stool Sample Collected ☐ yes ☐ no ☐ unk
Medical Evaluation ☐ yes ☐ no ☐ unk
Education Provided ☐ yes ☐ no ☐ unk

ADMINISTRATION

Remember to copy patient's name to the top of this page.

Initial report sent to OHS on ____/____/____

Completed by ______ Date ______ Phone ______

Case investigation sent to OHS on ____/____/____
