# Supplementary material for: Taenia solium Tapeworm Infection, Oregon, 2006–2009
Source: Emerg Infect Dis. 2011 Jun;17(6):1030–6. doi: 10.3201/eid1706.101397 (PMC3320238; doi:10.3201/eid1706.101397)
Supplement: Related texts

# Research article: *Taenia solium* Tapeworm Infection, Oregon, 2006–2009

**DOI:** 10.3201/eid1706.101397

**Authors:** Seth O’Neal, John Noh, Patricia Wilkins, William Keene, William Lambert, James Anderson, Jenifer Compton Luman, John Townes

**Affiliations:** Author affiliations: Oregon Health & Science University, Portland, Oregon, USA (S. O’Neal, W. Lambert, J. Anderson, J. Compton Luman, J. Townes);; Centers for Disease Control and Prevention, Atlanta, Georgia, USA (J. Noh, P. Wilkins);; Oregon Department of Human Services, Portland (W. Keene)

## Abstract

Neurocysticercosis (NCC) is a parasitic infection of the central nervous system caused by *Taenia solium* larval cysts. Its epidemiology in cysticercosis-nonendemic regions is poorly understood, and the role of public health institutions is unclear. To determine the incidence of NCC and to pilot screening of household contacts for tapeworms, we conducted population-based active surveillance in Oregon. We screened for *T. solium* infection by examining hospital billing codes and medical charts for NCC diagnosed during January 1, 2006–December 31, 2009 and collecting fecal and blood samples from household contacts of recent case-patients. We identified 87 case-patients, for an annual incidence of 0.5 cases per 100,000 general population and 5.8 cases per 100,000 Hispanics. In 22 households, we confirmed 2 additional NCC case-patients but no current adult intestinal tapeworm infections. NCC is of clinical and public health concern in Oregon, particularly among Hispanics. Public health intervention should focus on family members because household investigations can identify additional case-patients.

Neurocysticercosis (NCC) is a parasitic disease caused by central nervous system infection with *Taenia solium* larval cysts. It is the most common helminthic infection of the central nervous system and a leading cause of acquired epilepsy in Latin America, Southeast Asia, and central Africa (*1**,**2*). The disease also is increasingly of clinical and public health concern in the United States, primarily in immigrants and travelers from cysticercosis-endemic regions (*3**–**5*).

Cysticercosis is acquired through fecal–oral transmission of tapeworm eggs shed in the feces of a human carrying intestinal tapeworms. Ingested eggs release oncospheres, which invade the intestinal mucosa and disseminate throughout the body to form larval cysts. NCC occurs when cysts develop in the central nervous system and is the primary source of illness and death (*6*). The tapeworm’s complete life cycle occurs in regions with poor sanitary infrastructure, where foraging pigs have access to human feces. Most NCC cases in the United States probably were acquired in cysticercosis-endemic areas by immigrants or travelers who entered the United States already infected with cysts (*3*). However, immigrants and travelers also can harbor intestinal tapeworms, and domestic transmission of NCC does occur (*7**,**8*).

Few states require reporting of cysticercosis; thus, population-based epidemiologic data in the United States are limited. Even in jurisdictions that require reporting, the clinical nature of NCC diagnosis complicates surveillance efforts because no single laboratory test definitively establishes the diagnosis. Surveillance therefore relies on clinician or institutional reporting. In 1989, California became the first state to require reporting; 112 cysticercosis cases were reported during the first year, for a crude annual incidence of 1.5 cases per 100,000 Hispanics (*9*). A retrospective case-series from Oregon based on hospital discharge diagnoses during 1995–2000 estimated an annual incidence of 0.2 cases per 100,000 general population and 3.1 cases per 100,000 Hispanics (*10*). In 5 cases, no exposure to a cysticercosis-endemic area was documented, which suggests the possibility of local transmission.

Oregon adopted administrative rules for *T. solium* reporting in 2002 after the coroner’s examination implicated hydrocephalus secondary to obstructing ventricular cysts in 2 unexplained deaths (*10*). However, no subsequent efforts were undertaken to stimulate passive reporting or to actively find unreported cases. As a result, only 7 NCC cases, all in Hispanics, were reported to public health officials during the first 5 years of reporting. Oregon has a rapidly growing Hispanic population, which currently represents 11% of the total population. Approximately half of all Oregon Hispanics report birth outside the United States (*11*). In the context of an increasing population at risk, the small number of passively reported cases suggests inadequate surveillance.

Identification and treatment of tapeworm carriers in the United States could prevent additional NCC cases. However, intestinal tapeworm infection produces few symptoms, and the prevalence is typically <1%–2%, even in regions where cysticercosis is endemic (*12*). During the 1980s, Los Angeles (LA) County, California, adopted a program of screening for tapeworm carriers with some success. By screening household members of NCC case-patients using light microscopy on fecal samples, the county identified an intestinal tapeworm carrier in 7% of its overall investigations and in 22% of investigations involving domestically acquired NCC (*13*). Improved screening methods have been developed in the interim, including an ELISA for *Taenia* sp. coproantigens in feces and an enzyme-linked immunoelectrotransfer blot (EITB) for serum antibodies against *T. solium* tapeworm (*14**,**15*). Serologic methods are desirable because they are specific to *T. solium* intestinal infection and highly sensitive (99%) and avoid the collection and processing of potentially infectious feces (*15*).

Our objective was to evaluate the utility of public health surveillance for *T. solium* infection in Oregon. We implemented population-based active surveillance to determine the incidence of cysticercosis. We also piloted screening specifically for additional *T. solium* infection among affected households by using a combination of symptom screening, laboratory analysis of fecal and serum specimens, and radiographic imaging.

## Methods

### Case Definition and Surveillance Period

We used the cysticercosis case definition in State of Oregon communicable disease investigative guidelines (www.oregon.gov/DHS/ph/acd/reporting/guideln/taeniasis_cyst_guideline.pdf). This definition classifies cases as confirmed, presumptive, or suspected according to published consensus criteria (*2*). Because clinical criteria for a definitive diagnosis (pathologic specimen, radiographic imaging demonstrating the scolex or direct visualization of the parasite on fundscopic eye examination) are seldom available, a presumptive diagnosis is common. We defined cases as infection diagnosed initially during January 1, 2006–December 31, 2009. Cases in non-Oregon residents were excluded. The Institutional Review Boards at Oregon Health & Sciences University and the State of Oregon Public Health Division reviewed and approved this study.

### Case Ascertainment

During the 2009 calendar year, we requested quarterly reports of International Classification of Diseases, 9th Revision, billing codes for cysticercosis (123.1) from all Oregon hospital systems. The first request included identification of historical cases going back to January 1, 2006; subsequent reports included only cases for the current quarter. All hospitals reported inpatient admissions, and those with integrated electronic medical records systems reported outpatient visits as well. To identify additional cases, we queried the main regional reference laboratory for cysticercosis serologic testing in Oregon. We also searched Oregon vital statistics data for deaths related to cysticercosis under all listed causes of death, both by diagnosis code and by keyword (i.e., cysticercosis, neurocysticercosis, and taenia). We obtained medical charts for all reported cases to verify the diagnosis and to extract clinical and epidemiologic data.

To stimulate passive reporting, we sent a letter to clinicians likely to diagnose NCC, including migrant worker health providers, radiologists, pathologists, neurologists, neurosurgeons, and infectious disease and emergency department physicians. We also distributed a newsletter about *T. solium* infection and reporting requirements to all licensed Oregon physicians.

### Household Investigations

Persons with confirmed and presumptive cases diagnosed after July 1, 2008, were eligible for household investigation. After obtaining informed consent from the case-patient and all household contacts, the study physician used a standard interview tool to gather demographic, clinical, and epidemiologic data (Technical Appendix). From each participant, we collected 1 fecal sample preserved in 10% formalin and a finger-stick blood sample on quantifiable filter paper preserved in StabilZyme Select (SurModics; Eden Prairie, MN, USA) stabilizer. We offered noncontrast computed tomography (CT) scan of the head to any household contact with clinical history of seizures or severe or chronic headaches or with any positive finding from laboratory tests.

### Laboratory Methods

Laboratory processing was conducted at the Centers for Disease Control and Prevention Parasitology Diagnostics Laboratory (Atlanta, GA, USA). Fecal samples were examined by light microscopy for *Taenia* spp. eggs or proglottids and by ELISA for *Taenia* spp. coproantigens (*14*). Serum samples were analyzed by EITB for antibodies against *T. solium* cysts (EITB lentil lection–bound glycoprotein) and against *T. solium* adult tapeworms (recombinant EITB [rEITB]) (*15**,**16*). The EITB lentil lection–bound glycoprotein uses a semipurified fraction of homogenized *T. solium* cysts containing 7 *T. solium* glycoprotein antigens (*16*). The rEITB for taeniasis is based on baculovirus expression–purified recombinant antigen rES33 (*15*). We defined active intestinal (adult) tapeworm infection by either a positive ELISA coproantigen or by *Taenia* spp. eggs or proglottids in the fecal sample. We interpreted a positive serum rEITB in participants with negative fecal findings to indicate cleared *T. solium* intestinal tapeworm infection.

### Data Analysis

Annual incidence rates were expressed as the number of cases per 100,000 population, with denominator estimates obtained from American Community Survey yearly estimates (*11*). We analyzed data using STATA version 10 (Stata Corp., College Station, TX, USA). Continuous variables were assessed by using either Kruskal-Wallis or Mann-Whitney tests for differences among or between groups of interest. We used the Fisher exact test to compare distributions of proportions or to examine association between pairs of categorical measures. All tests are 2-sided, with significance set at 0.05.

## Results

We found 143 unique reports with diagnosis code 123.1 for Oregon residents during the surveillance period. Of the 56 (39%) reports we excluded, insufficient chart information was available to verify diagnosis or incidence year for 18 cases, and 38 cases were diagnosed before 2006. Of the remaining 87 cases, 79 (91%) were identified through active surveillance, including 75 (86%) through hospital queries, 2 (2%) through laboratory queries, and 2 (2%) during household investigations. Eight (9%) cases were spontaneously reported by clinicians, of which 6 were from 1 infectious disease clinician in a tertiary care hospital.

Of the 87 remaining cases, 19 (22%) were confirmed, 53 (61%) presumptive, and 15 (17%) suspected. Confirmed and presumptive cases therefore accounted for 72 (83%) of the total. All case-patients had radiographic imaging of the head; 83 (95%) had a CT scan, and 52 (60%) had magnetic resonance imaging (MRI). Confirmed and presumptive case-patients were 8.5× more likely than suspected case-patients to have received an MRI (odds ratio 8.5, 95% confidence interval 2.0–50.2). Birth country and travel history were not recorded for 12 of the suspected case-patients; an epidemiologic link to a cysticercosis-endemic area would have changed the classification to presumptive in all 12. The other 3 suspected case-patients had radiologic evidence suggestive of cysticercosis or links to a cysticercosis-endemic area, but their symptoms could have been explained by other diagnoses. Suspected case-patients were more likely to be female (p = 0.04) and older (p<0.01) and to have calcified lesions (p<0.01) than were confirmed or presumptive case-patients (Table 1).

**Table 1:** Demographic and clinical characteristics of cysticercosis case-patients, Oregon, 2006–2009

Characteristic	No. confirmed/presumptive (%), n = 72	No. suspected (%), n = 15	p value*
Demographic†			
M	46 (64)	5 (33)	0.04
Foreign-born	47 (65)	4 (27)	1.0
Signs and symptoms			
Seizures	29 (40)	4 (27)	0.07
Headache	27 (38)	3 (20)	
Focal neurologic deficit	5 (7)	1 (7)	
Altered mental status	3 (4)	3 (20)	
Traumatic injury	2 (3)	2 (13)	
Other	6 (8)	2 (13)	
Lesions			
1	22 (31)	2 (13)	0.10
2–4	21 (29)	9 (60)	
>5	29 (40)	4 (27)	
Lesion location			
Parenchymal	61 (85)	15 (100)	0.49
Extraparenchymal	3 (4)	0	
Mixed	8 (11)	0	
Lesion stage			
Cystic only	22 (31)	1 (7)	0.01
Calcified only	32 (44)	13 (87)	
Mixed	18 (25)	1 (7)	

*By Fisher exact test. †Median age, y (interquartile range): confirmed/presumptive, 36 (28–43); suspected, 47 (35–66); p<0.01, Mann-Whitney test.

Of the 72 confirmed and presumptive case-patients, 41 (57%) were hospitalized at time of diagnosis. The median inpatient stay was 4 days (interquartile range [IQR] 3–9), accounting for a total of 292 hospital days during initial illness only. Of these 41 hospitalizations, intensive care was involved in 16 (39%). Suspected case-patients were less likely to receive treatment with antiparasitic drugs (p = 0.03) or corticosteroids (p = 0.03); otherwise, hospitalization and treatment did not differ between patients with confirmed or presumptive cases. No deaths occurred for which cysticercosis was listed as a contributing factor.

We excluded suspected cases from incidence calculations (Table 2). Sixty-nine (96%) cases occurred in Hispanics. Including the 12 suspected cases for whom epidemiologic data were unavailable would increase the estimated mean annual incidence to 0.6 cases per 100,000 Oregon residents and 6.7 per 100,000 Hispanic Oregon residents. More case reports occurred during the active study year (2009), but the number of reports was not significantly higher in 2009 than in previous years (p = 0.08).

**Table 2:** Incidence of confirmed and presumptive cysticercosis cases, Oregon, 2006–2009

Variable	2006, n = 14	2007, n = 18	2008, n = 14	2009, n = 26*	All cases, n = 72
Hispanic, no. (%)	13 (93)	18 (100)	14 (100)	24 (92)	69 (96)
Years since immigration, median (interquartile range)	8 (5–10)	6 (2–10)	10 (8–17)	10 (4–18)	9 (4–15)
Annual incidence					
Cases/100,000 overall population	0.4	0.5	0.4	0.7	0.5
Cases/100,000 Hispanic population	3.4	4.5	3.4	5.5	5.8

*Number of reported confirmed and presumptive cases during the active study year (2009) compared with prior years, p = 0.08 by Fisher exact test.

Country of birth was documented in the medical charts of 55 case-patients (Table 3). Three (5%) were US born; all were Hispanic. One was a 49-year-old man who denied any international travel; he had 1 obstructing fourth ventricular cyst confirmed by surgical pathology. Another US-born case-patient was a 24-year-old man with new-onset seizures, a single cystic parenchymal lesion found on MRI, and positive serologic test results for *T. solium* cysts. His only international travel included 1 week in Mexico 17 years before diagnosis. For both of these case-patients, family members reported ongoing travel to and from Mexico. Travel history was not available for the final US-born case-patient, a 57-year-old man with multiple parenchymal calcifications, seizures, and psychosis.

**Table 3:** Region and country of orgin for the 55 cysticercosis case-patients for whom information was known, Oregon, 2006–2009

Region	No. (%) case-patients	Country (no. case-patients)
Central America, Caribbean	47 (85)	Mexico (40), Guatemala (5), Nicaragua (1), Cuba (1)
North America	3 (5)	United States (3)
Southeast Asia	2 (4)	Myanmar [Burma] (1), Thailand (1)
South America	1 (2)	Ecuador (1)
Africa	1 (2)	Cameroon (1)
Europe	1 (2)	Germany (1)

Thirty-two confirmed or presumptive NCC cases were initially diagnosed after July 1, 2008, and were therefore eligible for household investigation. We investigated 22 (69%) cases (Table 4). Of the 10 cases that were not investigated, 7 patients could not be located with the contact information available in the chart, 2 were identified in other household investigations, and 1 was unable to provide informed consent because of psychosis. No case-patient refused the offer for household investigation. We found no significant difference between the 22 cases we investigated and the 10 we did not with respect to patient demographic or clinical characteristics. We did not identify any significant difference between the 22 investigated cases and the 40 confirmed or presumptive cases diagnosed before July 1, 2008. For investigated case-patients, median time since immigration to the United States was 10 years (IQR 6–14 years) and median time from last international travel to a cysticercosis-endemic country was 5 years (IQR 2–10 years).

**Table 4:** Demographic and clinical characteristics of case-patients with investigated and noninvestigated cysticercosis , Oregon, 2006–2009*

Characteristic†	Eligible for household investigation		No. (%) not eligible, n = 40	p value‡
	No. (%) investigated, n = 22	No. (%) not Investigated, n = 10		
Male sex	14 (64)	9 (90)	23 (58)	0.16
Lesions				
1	6 (27)	2 (20)	14 (35)	0.55
>1	16 (73)	8 (80)	26 (65)	
Lesion location				
Parenchymal	17 (77)	9 (90)	35 (88)	0.21
Extraparenchymal	2 (9)	1 (10)	0	
Mixed	3 (14)	0	5 (13)	
Lesion stage				
Cystic	6 (27)	1 (10)	15 (38)	0.15
Calcified	8 (36)	8 (80)	16 (40)	
Mixed	8 (36)	1 (10)	9 (23)	
EITB LLGP positive	9 (41)	–	–	

*EITB LLGP, enzyme-linked immunoelectrotransfer blot for antibodies against lentil lection–bound glycoprotein of *Taenia solium* cysts. †Median age, y (interquartile range): investigated, 31 (28–37); not investigated, 42 (35–57); not eligible, 35 (25–43); p = 0.15 by Kruskal-Wallis χ^2^ test. ‡By Fisher exact test.

A median of 6 (IQR 4–7) persons resided in each household. Of 111 total contacts, 79 (71%) were foreign born, and 41 (37%) reported international travel within the past 2 years. All fecal samples were negative by light microscopy and ELISA for coproantigen. One household contact had serum antibodies against *T. solium* adult tapeworms in 2 (9%) separate household contact investigations. In 1 household, the seropositive person was the brother of the index NCC case-patient. In the other household, the seropositive person was the husband of the index NCC case-patient. Nine case-patients (41%) and 1 (1%) household contact had circulating antibodies against *T. solium* cysts.

We offered head CT scans to 11 household contacts, 3 on the basis of positive serologic test results and 8 on clinical history. Of 9 who accepted, 2 had parenchymal calcifications consistent with NCC. One was a 7-year-old child from Myanmar (Burma) who had resettled with his family in Oregon 1 year earlier. He had a 3-year history of recurrent, untreated, generalized seizures that had not been reported to his physician. His mother was the household index case-patient; she sought care initially for severe headache and new-onset seizure; she had positive serologic test results for *T. solium* cysts and >20 parenchymal cystic lesions. The boy’s father had serum antibodies against *T. solium* intestinal tapeworm infection with negative results of fecal studies. The other NCC case-patient was an adult man from Mexico City, Mexico, with an occipital parenchymal calcification and chronic headaches; he had immigrated 21 years earlier and denied international travel since immigration. We found no other evidence of *T. solium* infection in his household, other than the original case-patient.

## Discussion

In Oregon, *T. solium* causes illness, particularly among the Hispanic population, which maintains ongoing contact with cysticercosis-endemic Latin America through immigration and travel. The mean annual incidence among Hispanics of 5.8 cases per 100,000 population is the highest documented rate within the United States, 4× the estimates from California in the mid-1980s, and 2× the previous estimate for Oregon (*9**,**10**,**13*). Although we documented no deaths directly resulting from cysticercosis, the morbidity and associated use of the health care system are high. Hospitalization at time of diagnosis was common, and intensive care was required in more than one third of hospitalizations. We did not quantify the health resource use related to treatment and follow-up of cases, but we noted surgical complications, shunt failure, and side effects from prolonged steroid use.

The relatively high incidence of cysticercosis in this study probably reflects increased case ascertainment rather than any increase in the underlying risk. Prior studies have relied primarily on hospital discharge data for case finding, which do not capture emergency department visits unless they result in inpatient admission. By requesting quarterly reports based on hospital billing codes, we were able to capture emergency department diagnoses. Many of these appear to have been less clinically severe, including uncomplicated new-onset seizures and headaches from calcified or nonobstructing cysts. In others, we found subsequent inpatient stays for treatment complications or clinical deterioration. Oregon’s comparatively high incidence could alternatively be explained by underlying migration patterns, specifically if preferential migration to Oregon occurred from the highly cysticercosis-endemic central Mexican highlands. However, we have no definitive evidence to either support or refute this hypothesis.

Despite improved case ascertainment, for several reasons the incidence reported in this study most likely underestimates the true incidence of NCC in Oregon. First, we excluded suspected cases from incidence calculations. Although clinical and demographic characteristics of patients with suspected cases were similar to those with confirmed and presumptive cases, a documented epidemiologic link to a cysticercosis-endemic area was not found in all medical charts. For most persons with suspected cases, epidemiologic evidence suggesting exposure to *T. solium* would have changed the classification to presumptive. Including these suspected cases increased the mean annual incidence to 6.7 cases per 100,000 persons among Hispanics. Second, although several hospital systems reported outpatient visits related to cysticercosis, most outpatient visits in the state were not captured. Because less clinically severe disease can be diagnosed and treated completely in the outpatient setting, we may have missed these cases. Finally, we suspect that underdiagnosis is common, particularly in patients seeking care for headache related to intermittent inflammation around degenerating or calcified parenchymal cysts. The threshold for obtaining neuroimaging in the primary care setting often is high for chronic or intermittent headaches. The prevalence and health-resource use of headache related to NCC have not been characterized in cysticercosis-endemic or -nonendemic areas.

Opportunity to prevent NCC within the United States is primarily limited to identifying and treating domestic carriers of *T. solium* tapeworms. The numerous reports of NCC among US-born persons who have never traveled implicate domestic exposure to *T. solium* eggs (*3**,**7**,**17**–**20*). We found only 1 such person during our surveillance period, although other population studies have described probable domestic transmission in 7%–10% of NCC cases (*9**,**13*). Although most infected foreign-born persons were likely to have acquired infection outside the United States, some foreign-born case-patients may have acquired their disease within the United States. We documented frequent travel to and from cysticercosis-endemic areas among NCC case-patients and their household members, which suggests ongoing risk for tapeworm acquisition.

Despite the use of highly sensitive methods for testing serum and feces, we were unable to detect current intestinal tapeworm infection by screening household members of NCC case-patients. However, our case definition for current adult tapeworm infection was conservative. We found 2 contacts with circulating serum antibodies and negative results of fecal analysis, and we chose to interpret this discordance as evidence of past but cleared infection. However, given the high sensitivity and specificity of the rEITB, the unknown duration of antibody persistence, and the fact that we tested just 1 stool sample, an alternate interpretation of this discordance might be active adult tapeworm infection with false-negative results of fecal analysis.

We may not have been able to replicate the prior success in LA County, California, where tapeworm carriers were identified in 7% of investigated households (*13*), for other reasons. First, our sample size in this pilot program was small, and chance alone could explain the difference. Second, cases in LA County were based on date of symptom onset rather than date of diagnosis. Because symptom onset can substantially predate diagnosis, the cases we investigated may have been systematically biased toward more remote exposure. We defined cases according to date of diagnosis because exact symptom onset can be difficult to determine, particularly for chronic or intermittent headaches. Third, substantial underlying differences in case-patients and household contacts could exist between Oregon and LA County. With LA County’s proximity to the Mexico border, case-patients and household members may have traveled outside the United States more recently or more frequently. In Oregon, the median time since immigration for case-patients was 4 years longer than the median time since immigration for case-patients in the LA County study. Similarly, the surveillance or investigation results from each study may not be generalizable to other states or other countries in which cysticercosis is not endemic. Finally, *T. solium* control efforts have been initiated in many areas of Latin America, and the underlying prevalence of tapeworm infection among immigrants from those regions might have decreased in the 20 years since the LA County program was implemented.

The strategy of routine screening for tapeworm carriers among household contacts of a person with symptomatic NCC may be inherently limited because of the long latency between exposure to *T. solium* eggs and development of symptoms. We did find evidence of past tapeworm infection and possible transmission to other household members. Specific clinical or demographic characteristics of an NCC case-patient might correlate with the presence of a tapeworm in a household member, such as young age, remote exposure in a cysticercosis-endemic area, and viable or multiple lesions. Our sample size was too small to evaluate the effectiveness of limiting investigations based on these variables.

Even though the role of public health in tapeworm screening to prevent domestic transmission remains unclear, the public health system has other functions related to *T. solium* infection. Primary among these is a focus on the health of household members who are at increased risk for *T. solium* infection. Improved selection criteria for household investigations may increase the likelihood of detecting current tapeworm infection. Early identification, referral, and surgical treatment of chronic headache caused by hydrocephalus could prevent serious complications. Education of household members also is crucial because they may travel frequently to and from cysticercosis-endemic areas. Recognition of the need to avoid eating undercooked pork and to maintain good hygiene can reduce infection among travelers. Finally, increasing clinician awareness about *T. solium* infection is a necessary public health function, particularly for clinicians who care for Hispanic and other immigrant populations. Public health intervention should focus on the health of household members and on increasing awareness of the disease among affected families and among clinicians.

## References

[1] Román G, Sotelo J, Del Brutto O, Flisser A, Dumas M, Wadia N, A proposal to declare neurocysticercosis an international reportable disease. Bull World Health Organ. 2000;78:399–406.10812740PMC2560715

[2] Del Brutto OH, Rajshekhar V, White AC Jr, Tsang VC, Nash TE, Takayanagui OM, Proposed diagnostic criteria for neurocysticercosis. Neurology. 2001;57:177–83.1148042410.1212/wnl.57.2.177PMC2912527

[3] Wallin MT, Kurtzke JF. Neurocysticercosis in the United States: review of an important emerging infection. Neurology. 2004;63:1559–64.1553423610.1212/01.wnl.0000142979.98182.ff

[4] Sorvillo FJ, DeGiorgio C, Waterman SH. Deaths from cysticercosis, United States. Emerg Infect Dis. 2007;13:230–5. 10.3201/eid1302.06052717479884PMC2725874

[5] Croker C, Reporter R, Mascola L. Use of statewide hospital discharge data to evaluate the economic burden of neurocysticercosis in Los Angeles County (1991–2008). Am J Trop Med Hyg. 2010;83:106–10. 10.4269/ajtmh.2010.09-049420595487PMC2912585

[6] García HH, Gonzalez AE, Evans CA, Gilman RH. *Taenia solium* cysticercosis. Lancet. 2003;362:547–56. 10.1016/S0140-6736(03)14117-712932389PMC3103219

[7] Schantz PM, Moore AC, Muñoz JL, Hartman BJ, Schaefer JA, Aron AM, Neurocysticercosis in an Orthodox Jewish community in New York City. N Engl J Med. 1992;327:692–5. 10.1056/NEJM1992090332710041495521

[8] Asnis D, Kazakov J, Toronjadze T, Bern C, Garcia HH, McAuliffe I, Neurocysticercosis in the infant of a pregnant mother with a tapeworm. Am J Trop Med Hyg. 2009;81:449–51.19706913

[9] Ehnert KL, Roberto RR, Barrett L, Sorvillo FJ, Rutherford GW III. Cysticercosis: first 12 months of reporting in California. Bull Pan Am Health Organ. 1992;26:165–72.1638281

[10] Townes JM, Hoffmann CJ, Kohn MA. Neurocysticercosis in Oregon, 1995–2000. Emerg Infect Dis. 2004;10:508–10.1510942410.3201/eid1003.030542PMC3322801

[11] United States Census Bureau. Oregon: selected population profile in the United States. Hispanic or Latino (of any race). 2008 American Community Survey 1-year estimates [cited 2010 May 1]. http://factfinder.census.gov

[12] Flisser A. Where are the tapeworms? Parasitol Int. 2006;55:S117–20. 10.1016/j.parint.2005.11.01816337430

[13] Sorvillo FJ, Waterman SH, Richards FO, Schantz PM. Cysticercosis surveillance: locally acquired and travel-related infections and detection of intestinal tapeworm carriers in Los Angeles County. Am J Trop Med Hyg. 1992;47:365–71.152415010.4269/ajtmh.1992.47.365

[14] Allan JC, Avila G, Garcia Noval J, Flisser A, Craig PS. Immunodiagnosis of taeniasis by coproantigen detection. Parasitology. 1990;101:473–7. 10.1017/S00311820000606862092303

[15] Levine MZ, Lewis MM, Rodriquez S, Jimenez JA, Khan A, Lin S, Development of an enzyme-linked immunoelectrotransfer blot (EITB) assay using two baculovirus expressed recombinant antigens for diagnosis of *Taenia solium* taeniasis. J Parasitol. 2007;93:409–17. 10.1645/GE-938R.117539427

[16] Tsang VC, Brand JA, Boyer AE. An enzyme-linked immunoelectrotransfer blot assay and glycoprotein antigens for diagnosing human cysticercosis (*Taenia solium*). J Infect Dis. 1989;159:50–9. 10.1093/infdis/159.1.502909643

[17] Mody R, Nield LS, Stauffer W, Kamat D. Seizures in a 20-month-old native of Minnesota: a case of neurocysticercosis. Pediatr Emerg Care. 2005;21:860–2. 10.1097/01.pec.0000190232.20233.4516340766

[18] Keane JR. Cysticercosis acquired in the United States. Ann Neurol. 1980;8:643. 10.1002/ana.4100806247212657

[19] Centers for Disease Control. Locally acquired neurocysticercosis—North Carolina, Massachusetts, and South Carolina, 1989–1991. MMWR Morb Mortal Wkly Rep. 1992;41:1–4.1727973

[20] Kruskal BA, Moths L, Teele DW. Neurocysticercosis in a child with no history of travel outside the continental United States. Clin Infect Dis. 1993;16:290–2. 10.1093/clind/16.2.2908443310

# Research article: *Taenia solium* Tapeworm Infection, Oregon, 2006–2009

**DOI:** 10.3201/eid1706.101397

**Authors:** Seth O’Neal, John Noh, Patricia Wilkins, William Keene, William Lambert, James Anderson, Jenifer Compton Luman, John Townes

**Affiliations:** Author affiliations: Oregon Health & Science University, Portland, Oregon, USA (S. O’Neal, W. Lambert, J. Anderson, J. Compton Luman, J. Townes);; Centers for Disease Control and Prevention, Atlanta, Georgia, USA (J. Noh, P. Wilkins);; Oregon Department of Human Services, Portland (W. Keene)

## Earning CME Credit

To obtain credit, you should first read the journal article. After reading the article, you should be able to answer the following, related, multiple-choice questions. To complete the questions and earn continuing medical education (CME) credit, please go to www.medscape.org/journal/eid. Credit cannot be obtained for tests completed on paper, although you may use the worksheet below to keep a record of your answers. You must be a registered user on Medscape.org. If you are not registered on Medscape.org, please click on the New Users: Free Registration link on the left hand side of the website to register. Only one answer is correct for each question. Once you successfully answer all post-test questions you will be able to view and/or print your certificate. For questions regarding the content of this activity, contact the accredited provider, CME@medscape.net. For technical assistance, contact CME@webmd.net. American Medical Association’s Physician’s Recognition Award (AMA PRA) credits are accepted in the US as evidence of participation in CME activities. For further information on this award, please refer to http://www.ama-assn.org/ama/pub/category/2922.html. The AMA has determined that physicians not licensed in the US who participate in this CME activity are eligible for *AMA PRA Category 1 Credits*™. Through agreements that the AMA has made with agencies in some countries, AMA PRA credit is acceptable as evidence of participation in CME activities. If you are not licensed in the US and want to obtain an AMA PRA CME credit, please complete the questions online, print the certificate and present it to your national medical association.

## Article Title: *Taenia solium* Tapeworm Infection, Oregon, 2006–2009

## CME Questions

**1. On the basis of the current population surveillance study by Dr. O’Neal and colleagues, which of the following statements about the epidemiology of cysticercosis in Oregon is most likely correct?**A. Cysticercosis is not a significant clinical or public health disease in OregonB. Among Hispanics, the annual incidence of cysticercosis is at least 5.8/100,000 populationC. The current incidence of cysticercosis among Hispanics has not changed since the prior estimate for OregonD. The observed incidence in this study likely overestimates the true incidence of NCC in Oregon**2. You are a public health official in Oregon planning for needed services related to cysticercosis and NCC. On the basis of O’Neal and colleagues’ study, which of the following statements about morbidity and mortality is most likely correct and therefore likely to be a factor during planning?**A. The mortality rate in this study was 10%B. Hospitalization at time of diagnosis was rareC. There were no hospitalizations requiring intensive careD. Surgical complications, shunt failure, and adverse events from prolonged steroid use were noted**3. As the public health official described in question 2, you are now considering appropriate goals of public health interventions for cysticercosis. Which of the following statements is most likely correct on the basis of the current study?**A. Improved selection criteria for household investigations may increase the likelihood of detecting current taeniasis infectionB. Public health intervention should focus on the health of workplace contactsC. Affected families are already likely to understand how to prevent transmissionD. Clinicians already have a high index of suspicion for this disease

## Activity Evaluation

**Table:** 

1. The activity supported the learning objectives.				
Strongly Disagree				Strongly Agree
1	2	3	4	5
2. The material was organized clearly for learning to occur.				
Strongly Disagree				Strongly Agree
1	2	3	4	5
3. The content learned from this activity will impact my practice.				
Strongly Disagree				Strongly Agree
1	2	3	4	5
4. The activity was presented objectively and free of commercial bias.				
Strongly Disagree				Strongly Agree
1	2	3	4	5

## Supplementary Material

Technical Appendix Interview tool used to gather demographic, clinical, and epidemiologic data from study participants.
